# Abnormally increased carotid intima media-thickness and elasticity in patients with Morquio A disease

**DOI:** 10.1186/s13023-020-1331-y

**Published:** 2020-03-17

**Authors:** Raymond Y. Wang, Kyle D. Rudser, Donald R. Dengel, Nicholas Evanoff, Julia Steinberger, Nina Movsesyan, Robert Garrett, Katherine Christensen, Deborah Boylan, Stephen R. Braddock, Marwan Shinawi, Qi Gan, Adriana M. Montaño

**Affiliations:** 1 Division of Metabolic Disorders, CHOC Children’s Specialists, Orange, CA USA; 2 Department of Pediatrics, University of California-Irvine School of Medicine, Orange, CA USA; 3 Division of Biostatistics, School of Public Health, University of Minnesota, Minneapolis, MN USA; 4 School of Kinesiology, College of Education and Human Development, University of Minnesota, Minneapolis, MN USA; 5 Department of Pediatrics, University of Minnesota Medical School, Minneapolis, MN USA; 6 Research Institute, CHOC Children’s Hospital, Orange, CA USA; 7 Department of Radiology, School of Medicine, Saint Louis University, Saint Louis, MO USA; 8 Department of Pediatrics, Doisy Research Center, School of Medicine, Saint Louis University, St. Louis, MO USA; 9 SSM Cardinal Glennon Children’s Hospital, Saint Louis, MO USA; 10 Department of Pediatrics, Division of Genetics and Genomic Medicine, Washington University School of Medicine, Saint Louis, MO USA; 11 Department of Biochemistry and Molecular Biology, School of Medicine, Saint Louis University, Saint Louis, MO USA

**Keywords:** Morquio A disease, Mucopolysaccharidosis IVA, Cardiovascular disease, Carotid ultrasound, Intima media thickness

## Abstract

**Background:**

Cardiovascular disease frequently causes morbidity and mortality in mucopolysaccharidoses (MPS); however, cardiovascular anatomy and dysfunction in MPS IVA (Morquio A disease) is not well described. Consequently, the study aimed to compare carotid artery structure and elasticity of MPS IVA patients with other MPS patients and healthy control subjects, and quantitate frequency of MPS IVA cardiac structural and functional abnormalities.

**Methods:**

Prospective, multi-center echocardiogram and carotid ultrasound evaluations of 12 Morquio A patients were compared with other MPS and healthy control subjects. Average differences between groups were adjusted for age, sex, and height with robust variance estimation for confidence intervals and *P*-values.

**Results:**

Morquio A patients demonstrated significantly higher (*P* < 0.001) adjusted carotid intima-media thickness (cIMT), mean (SD) of 0.56 mm (0.03) compared to control subjects, 0.44 mm (0.04). The Morquio A cohort had significantly greater adjusted carotid elasticity (carotid cross-sectional compliance + 43%, *P* < 0.001; carotid incremental elastic modulus − 33%, *P* = 0.003) than control subjects and other MPS patients. Aortic root dilatation was noted in 56% of the Morquio A cohort, which also had highly prevalent mitral (73%) and aortic (82%) valve thickening, though hemodynamically significant valve dysfunction was less frequent (9%).

**Conclusions:**

Increased carotid elasticity in Morquio A patients is an unexpected contrast to the reduced elasticity observed in other MPS. These Morquio A cIMT findings corroborate MPS IVA arterial post-mortem reports and are consistent with cIMT of other MPS. Aortic root dilatation in Morquio A indicates arterial elastin dysfunction, but their carotid hyperelasticity indicates other vascular intima/media components, such as proteoglycans, may also influence artery function. Studying MPS I and IVA model systems may uniquely illuminate the function of glycosaminoglycan-bearing proteoglycans in arterial health.

## Background

Mucopolysaccharidosis IVA (MPS IVA, Morquio A disease, MIM#253000) is an autosomal recessive lysosomal storage disorder caused by a deficiency of N-acetylgalactosamine-6-sulfate sulfatase (GALNS). This deficiency results in excessive systemic storage of the glycosaminoglycans (GAGs) keratan sulfate (KS) and chondroitin 6-sulfate (C6S) in tissues and organs [1]. Morquio A patients have coarse facial features, hearing and vision loss, systemic skeletal dysplasia, disproportionate short-trunked short stature, joint abnormalities, hepatomegaly, pulmonary compromise, and valvular heart disease [2]. Though the focus is often on skeletal deformities, cardiac anomalies have been reported as the first sign of Morquio A as early as 3.6 months of age [3]. Cardiac anomalies observed in Morquio A include mitral and/or aortic insufficiency, aortic stenosis, tricuspid regurgitation, thickened interventricular septum, and hypertrophic cardiomyopathy [3–6]. Echocardiography indicates that cardiac lesions worsen in patients [3]. An observational study that included 54 Morquio A patients showed enlarged aortic roots, thickened left ventricles, smaller end-diastolic dimensions of the heart, reduced stroke volumes and impaired diastolic filling patterns [7]. Effect of enzyme replacement therapy (ERT) seemed negligible after a median follow up of 2.6–3.0 years [5, 7]. In addition, histopathology in a 20-year-old patient revealed foam cells and macrophages in aorta, aortic, mitral and tricuspid valves [8]. Cells in the aortic intima and media were vacuolated with GAGs, and elastin fibers were attenuated and fragmented. The intima was thickened by GAG accumulation, atherosclerotic plaques were found, and the aortic valve was hypertrophic [8].

Studies show carotid intima-media thickness (cIMT) could be a marker of atherosclerosis and cardiovascular risk in patients [9]. cIMT and carotid stiffness have been consistently increased in MPS patients when compared to age-matched controls [10, 11]. To date, no study explores cIMT in Morquio A. This study aimed to compare cIMT and carotid stiffness of Morquio A patients with other MPS patients and healthy controls.

## Results

### Morquio A carotid intima media-thickness

Details regarding the control and non-Morquio MPS cohorts have been published [11, 12]. The Morquio A cohort (*n* = 12) was 58% male and 42% female (Table 1). Patient 2 had a follow-up study visit with carotid ultrasound 16 months after initiating enzyme replacement. Data from this subsequent visit are included in reporting and statistical analysis, resulting in 13 data points (Table 2). Although the age at visit is slightly older (mean ± SD), (14.0 ± 10 years) due to the inclusion of four adults, mean height of the Morquio A cohort (108 ± 21.89 cm) was much less than the control (155 ± 18.30 cm) and non-Morquio MPS (132 ± 16.55 cm) cohorts. The mean Morquio A cohort cIMT was 0.56 ± 0.03 mm, while the non-Morquio MPS cohort cIMT was 0.56 ± 0.06 mm, and the control cohort cIMT was 0.44 ± 0.04 mm. The mean Morquio A cIMT was 0.12 mm greater than the mean control cohort cIMT, an increase of 25% (See Table 3 for detailed comparisons).

**Table 1:** Clinical, biochemical, and molecular characteristics of patients with Morquio A disease who participated in study

Patient	Age	Gender	Race, ethnicity	Country of origin	GALNS activity	Total Glycosaminoglycans	*GALNS* mutation	Height	Phenotype
	(years)					NR (149.4–207.9 ng/mg Cre)		(z-score)	
1	12.7	Female	Caucasian	Iraq	40 pmol/mg/h	2799.55 ng/mg Cre	c.230 C > G / c.230 C > G	−7.0	Severe
					(NR 400–2000)				
2	8.3	Male	Caucasian	Iraq	28 pmol/mg/h	1924.52 ng/mg Cre	c.230 C > G / c.230 C > G	−6.1	Severe
					(RR 400–2000)				
3	38.4	Male	Caucasian	US	0.06 nmol/mg/h	1158.20 ng/mg Cre	c.1353 G > T / c.1353 G > T	−3.4	Attenuated
					(NV 0.95)				
4	6.4	Female	Chinese	China	0.58 nmol/17 h/ mg	2266.21 ng/mg Cre	c.106_111delCTGCTC / c.953 T > G	−6.0	Severe
					(RR 42.3–441.9)				
5	18.3	Male	Hispanic	Mexico	Not Available	479.51 ng/mg Cre	c.1156C > T / 32 kb del	−8.11	Severe
6	3.9	Male	Hispanic	US	Not Available	1753.43 ng/mg Cre	c.901G > T / c.1520G > T	−3.73	Severe
7	8.5	Female	Caucasian	US	Deficient	Not Available	c.239C > T /?	−6.77	Severe
8	4.6	Female	Caucasian	US	Not Available	Not Available	c.239C > T /?	−6.34	Severe
9	25.9	Male	Hispanic	US	Deficient	Qualitatively Elevated	Not Available	−6.09	Severe
10	22.2	Male	Hispanic	US	7000 U	Elevated	c.1156C > T / c.924 T > G + c.930G > C	−9.0	Severe
					(RR > 40,000)				
11	10.4	Female	Hispanic / Caucasian	US	0.05 U	Qualitatively Elevated	c.776G > A / c.1520G > T	−2.23	Attenuated
					(RR > 0.7)				
12	16.1	Male	Hispanic	US	0 nmol / 17 h / mg	8.4 mg/mmol Creat	c.281G > A / c.1156C > T	−4.27	Severe
					(RR > 92)	(NR < 6.5)

**Table 2:** Patient characteristics

Covariate	Control	Other MPS	Morquio A
	(*n* = 560)	(*n* = 33)	(*n* = 13)
Male	301 (53.8%)	22 (67%)	8 (61%)
Female	259 (46.2%)	11 (33%)	5 (39%)
Age at visit (years)	13.14 (4.01)	12.41 (4.74)	13.98 (10.0)
SBP (mm Hg)	106 (10.42)	107 (10.79)	103 (12.8)
DBP (mm Hg)	58.00 (7.78)	55.21 (13.54)	70.6 (12.2)
Pulse Pressure (mm Hg)	48.12 (9.49)	51.97 (11.81)	31.92 (6.53)
Height (cm)	155 (18.30)	132 (16.55)	108 (21.89)
cIMT (mm)	0.44 (0.04)	0.56 (0.06)	0.56 (0.03)
cCSD (%)	32.03 (8.35)	28.16 (15.74)	25.37 (8.81)
cCSC1 (mm^2^ · mm Hg^−1^)	0.16 (0.05)	0.14 (0.08)	0.22 (0.07)
cIEM (mm Hg)	951 (379.84)	1355 (811.82)	772 (371)

Values presented are mean (sd) or *n* (%) where indicated. The “Other MPS” cohort is composed of 17 MPS I, 9 MPS II, 4 MPS IIIA, and 3 MPS VI patients. The Morquio A data includes one patient (#2), who underwent two carotid ultrasounds 16 months apart

*SBP* Systolic Blood Pressure, *DBP* Diastolic Blood Pressure, *cIMT* Carotid Intima-Media Thickness, *cCSD* Carotid Cross-sectional Distensibility, *cCSC1* Carotid Cross-sectional Compliance, *cIEM* Carotid Incremental Elastic Modulus

**Table 3:** Ratio comparisons between mean carotid measures of Morquio A patients, non-Morquio MPS patients (“Other MPS”), and pediatric control groups adjusted for sex, age, and height

Vascular Measure	Covariate	Ratio (95% CI)	*P*-value
	Control vs. Morquio A	0.80 (0.76, 0.84)	***< 0.001***
	Other MPS vs. Morquio A	1.00 (0.95, 1.05)	0.956
cIMT (mm)	Female (vs. Male)	0.98 (0.97, 1.00)	***0.038***
	Age (per 10 yr)	1.03 (0.99, 1.06)	0.114
	Height (per 10 cm)	1.00 (0.99, 1.01)	0.931
	Control vs. Morquio A	1.07 (0.88, 1.30)	0.521
	Other MPS vs. Morquio A	1.02 (0.80, 1.30)	0.875
cCSD (%)	Female (vs. Male)	1.01 (0.97, 1.05)	0.668
	Age (per 10 yr)	0.71 (0.64, 0.79)	***< 0.001***
	Height (per 10 cm)	1.04 (1.02, 1.06)	***< 0.001***
	Control vs. Morquio A	0.70 (0.57, 0.87)	***< 0.001***
cCSC1	Other MPS vs. Morquio A	0.61 (0.47, 0.79)	***< 0.001***
(mm^2^ · mm Hg^−1^)	Female (vs. Male)	0.95 (0.90, 1.00)	***0.045***
	Age (per 10 yr)	0.83 (0.74, 0.93)	***0.001***
	Height (per 10 cm)	1.01 (0.98, 1.04)	0.502
	Control vs. Morquio A	1.50 (1.15, 1.96)	***0.003***
	Other MPS vs. Morquio A	2.18 (1.63, 2.92)	***< 0.001***
cIEM (mm Hg)	Female (vs. Male)	0.95 (0.90, 1.01)	0.121
	Age (per 10 yr)	1.48 (1.30, 1.70)	***< 0.001***
	Height (per 10 cm)	0.99 (0.95, 1.02)	0.364

Ratios less than 1.00 indicate the first comparator is less than the second comparator; ratios greater than 1.00 indicate the first comparator is greater than the second comparator. Significant comparisons are highlighted in ***bold***

Comparison of mean carotid measures in all cohorts were adjusted for sex, age, and height, known covariates of cIMT and carotid stiffness (Table 3). The covariate-adjusted mean cIMT of the Morquio A patients was significantly greater than that of the control cohort (*P* < 0.001) and comparable to the non-Morquio MPS patient cohort (*P* = 0.866).

### Carotid artery compliance and distensibility assessment

In contrast to the non-Morquio MPS cohort, which had significantly increased carotid stiffness compared to controls, the Morquio A cohort demonstrated significantly increased carotid elasticity compared to controls. Following adjustment for age, sex, and height, the Morquio A cohort had significantly higher cCSC1 (+ 0.07 mm^2^ · mm Hg^− 1^; 95% confidence interval [+ 0.03 to + 0.11] mm^2^ · mm Hg^− 1^; *P* = 0.001) and lower cIEM (− 294 mmHg; [− 503 to − 85.7] mm Hg; *P* = 0.006) than the control patient cohort. This reflects a 43% increase [+ 15% to 75%] in cross-sectional compliance, and a 33% [− 49% to − 13%] reduction in incremental elastic modulus, of the Morquio A patients when compared to the control cohort. Carotid cross-sectional distensibility did not differ significantly between Morquio A and control cohorts (− 7.5%; [− 30% to + 12%]; *P* = 0.52).

The Morquio A cohort also had significantly higher cCSC (+ 0.09 mm^2^ · mm Hg^− 1^; [+ 0.05 to + 0.14] mm^2^ · mm Hg^− 1^; *P* < 0.001) and lower cIEM (− 681 mmHg; [− 984 to − 379] mm Hg; *P* < 0.001) than the non-Morquio MPS cohort, indicating increased Morquio A carotid elasticity compared to MPS patients who did not have Morquio A syndrome. The mean Morquio A cross-sectional compliance was 64% greater, and the incremental elastic modulus 54% reduced, compared to the non-Morquio MPS patients. Overall, age was the strongest and most consistent significant co-variate aside from MPS diagnosis for increasing carotid stiffness, with reduced cCSD: 29% decline per 10 years, *P* < 0.001; cCSC1: 17% decline per 10 years, *P* < 0.001; and increased cIEM: + 48% increase per 10 years, *P* < 0.001).

### Treatment effects upon carotid measurements

No clear differences in cIMT or other measurements were observed in the two studies obtained from patient 2 (first measurement pre-ERT and second measurement 14 months post-ERT). To further examine effects of treatment upon carotid structure and function, a comparison was made between the patients who were receiving ERT at the time of study, and those who were not receiving ERT. There were no significant differences in cIMT (0.00 mm, 95% confidence interval − 0.03 to + 0.03 mm; *P* = 0.92), cCSD (− 2.20%, [− 8.91% to + 4.51%]; *P* = 0.52), or cCSC (− 0.03 mm^2^ · mm Hg^− 1^, [− 0.08 to + 0.02] mm^2^ · mm Hg^− 1^; *P* = 0.28) between the Morquio A patients currently treated with ERT and those not treated with ERT adjusting for sex, age, and height (Table 4). Morquio A patients treated with ERT had significantly increased cIEM compared to those not treated (+ 137, [+ 2.44 to + 271.29] mm Hg; *P* = 0.046). For this comparison, height was a significant co-variate for carotid stiffness, with reduced cCSD: − 2.98% / 10 cm, *P* < 0.001; cCSC1: − 0.02 mm^2^ · mm Hg^− 1^/ 10 cm, *P* < 0.001; and increased cIEM: + 131 mmHg / 10 cm, *P* < 0.001).

**Table 4:** Comparisons between mean carotid measures of Morquio A patients treated with Enzyme Replacement Therapy (ERT) and those not treated with ERT

Score	Covariate	Difference (95% CI)	*P*-value
cIMT	ERT (vs. not)	0.00 (−0.03, 0.03)	0.916
(mm)	Female (vs. Male)	−0.01 (−0.05, 0.02)	0.402
	Age (per 10 yr)	0.01 (− 0.01, 0.02)	0.587
	Height (per 10 cm)	0.00 (−0.01, 0.00)	0.408
cCSD	ERT (vs. not)	−2.20 (−8.91, 4.51)	0.520
(%)	Female (vs. Male)	0.14 (−8.06, 8.34)	0.973
	Age (per 10 yr)	1.31 (−4.23, 6.86)	0.643
	Height (per 10 cm)	−2.98 (−5.38, −0.58)	***0.015***
cCSC1	ERT (vs. not)	−0.03 (− 0.08, 0.02)	0.279
(mm^2^	Female (vs. Male)	−0.03 (− 0.09, 0.04)	0.465
· mm Hg^−1^)	Age (per 10 yr)	0.01 (−0.03, 0.05)	0.686
	Height (per 10 cm)	−0.02 (− 0.04, − 0.01)	***0.005***
cIEM	ERT (vs. not)	136.86 (2.44, 271.29)	***0.046***
(mm Hg)	Female (vs. Male)	14.88 (− 145.24, 175.00)	0.855
	Age (per 10 yr)	34.26 (− 105.85, 174.38)	0.632
	Height (per 10 cm)	130.82 (80.41, 181.23)	***< 0.001***

Analyses are adjusted for sex, age, and height. Significant comparisons are highlighted in ***bold***. Height is a significant influence upon cCSD, cCSC1, and cIEM

### Cardiac structure and function

The Morquio A cohort had high occurrence of mitral (72%; 8 of 11, 95% confidence interval 39 to 93%) and aortic valve (82%; 9 of 11, [48 to 97%]) thickening (Table 5). Though abnormal valve anatomy was frequent, dysfunction was not severe. Trivial mitral valve regurgitation was noted in 18% (2 of 11), [3 to 52%] of Morquio patients. There did not appear to be an age predisposition for mitral valve dysfunction, as the two individuals were 3.9 and 8.5 years of age and were noted to have “thickened” and “mildly dysplastic” mitral valves, respectively. Aortic valve regurgitation was present in 27% of (3 of 11), [7 to 61%] Morquio A patients. Two patients had trivial aortic regurgitation; they were aged 8.5 and 16.1 years. One patient had combined regurgitation of both mitral and aortic valves. One patient, aged 22.3 years, had more prominent aortic regurgitation that had resulted in mild left ventricular dilatation. No patients demonstrated abnormal cardiac contractility or function. Aortic root dilatation, defined as a measurement at the level of the Sinuses of Valsava exceeding 2 standard deviations above the mean, was also frequent in the Morquio A population (56%; 5 of 9, [23 to 85%]).

**Table 5:** Echocardiographic and demographic parameters of the Morquio A cohort indicating high prevalence of mitral and aortic valve pathology, as well as aortic root dilatation. Aortic root Z-scores that indicate aortic root dilatation are highlighted in **bold.** n.a.; not analyzed

Patient	Mitral Valve		Aortic Valve		LV Size / Function	Aortic Root Z-Score
	Anatomy	Function	Anatomy	Function		
1	Mildly Thickened	Normal	Normal	Normal	Normal	n.a.
2	Mildly Thickened	Normal	Mildly Thickened	Normal	Normal	n.a.
3	Normal	Normal	Normal	Normal	Normal	+ 1.11
4	Mildly Thickened	Normal	Mildly Thickened	Normal	Normal	**+ 2.41**
5	n.a.	n.a.	n.a.	n.a.	n.a.	n.a.
6	Thickened	Trivial Regurgitation	Thickened	Normal	Normal	**+ 3.28**
7	Mild Dysplasia	Trivial Regurgitation	Thickened	Trivial Regurgitation	Normal	+ 1.97
8	Thickened	Normal	Thickened	Normal	Normal	+ 1.06
9	Mildly Thickened	Normal	Mildly Thickened	Normal	Normal	**+ 4.62**
10	Normal	Normal	Cusp Asymmetry	Moderate Regurgitation	Mild dilatation	**+ 4.92**
11	Minimally Thickened	Normal	Minimally Thickened	Normal	Normal	+ 0.43
12	Normal	Normal	Slightly Thickened	Trivial Regurgitation	Normal	**+ 2.43**
	73% Thickened	18% Regurgitation	82% Abnormal	27% Regurgitation	9% LV Dilatation	56% with Dilatation
	27% Normal	82% Normal	18% Normal	73% Normal	91% Normal	44% Normal

### Patient brief summaries

#### Patient 1

Iraqi Caucasian girl, diagnosed with Morquio A at 10.6 years of age. Sequencing of *GALNS* showed that she was homozygous for the pathogenic variant c.230C > G (p.P77R). Study echocardiography and carotid ultrasounds were performed at 11.2 and 12.7 years of age. Her first Morquio A-related symptom was kyphosis at 4 months of age; she suffered from frequent upper respiratory infections and was unable to walk by 5 years of age. Physical examination identified coarse facial features, corneal clouding, macrocephaly, short nose, poor dentition with wide spaced teeth, and mandibular prominence. Skeletal dysplasia was evident, with short stature, short trunk, kyphosis, lordosis, pectus carinatum, lower rib flaring, short neck with decreased rotation and flexion, genu valgum, platyspondyly, and marked laxity in wrists and fingers leading to difficulties with fine and gross motor tasks. She had contractures at knees, hips and ankles, canal stenosis, cervical cord compression, myelopathy and cervical instability. There was progressive weakness and difficulty breathing at rest. Patient had obstructive sleep apnea. The echocardiogram showed mild thickening of the mitral valve, no significant stenosis, no significant ventricular hypertrophy and normal biventricular systolic function. Patient had her first recombinant human GALNS (rhGALNS) enzyme infusion at 11.6 years old without any complications. At 11.7 years, she underwent tracheostomy, cervical fusion and decompression. At 12.5 years, bilateral sensorineural hearing loss was diagnosed.

#### Patient 2

Iraqi Caucasian boy; he is the younger sibling of patient 1 and was diagnosed with Morquio A at 6.2 years of age with homozygosity of the c.230C > G (p.P77R) family variant. Study echocardiography and carotid ultrasounds were performed at 7.9 and 9.2 years of age. Physical examination identified macrocephaly, coarse facial features, corneal clouding, poor dentition, gingival hypertrophy, and mandibular prominence. He had skeletal dysplasia with short stature, short and stiff neck, platyspondyly, kyphosis at the thoracolumbar junction, anterior beaking in vertebral bodies, oar-shaped deformity of ribs, deformed femoral heads, flat acetabular roofs, bilateral coxa valga, genu valgum, pectus carinatum, wrist deformity, progressive joint laxity, difficulty with fine and gross motor tasks, and diminished motion of shoulders. He had difficulty walking, a gait problem and general weakness. At 7 years, atlantoaxial subluxation with hypoplastic C2 odontoid process was confirmed, with subsequent occipital-cervical instability, myelopathy, and decline of ambulation. Echocardiogram showed very mild thickening of aortic and mitral valves, no significant valvar stenosis, regurgitation or ventricular hypertrophy. He had normal biventricular systolic function. Pulmonary studies suggested a narrowed oropharynx, impaired cough clearance, severe respiratory muscle weakness, neuromuscular weakness, daytime hypersomnolence, upper airway obstruction, restrictive lung disease with shortness of breath and obstructive sleep apnea, and multiple upper respiratory infections. First rhGALNS enzyme replacement therapy (ERT) occurred at 7.2 years without complications. He reported increased stamina after ERT, but subsequently developed a rash after enzyme infusions.

#### Patient 3

American Caucasian man of Middle Eastern ancestry. He was diagnosed at 12 years of age. Molecular studies identified the *GALNS* mutations c.1353G > T / c.1353G > T (p.R451S/R451S). Study echocardiography and carotid ultrasound were performed at 38.4 years of age. His skeletal dysplasia includes decreased bone density, generalized platyspondyly, bullet-shaped vertebra at the thoracolumbar junction, and generalized central canal stenosis of the cervical spine. He also had anterior atlantoaxial instability with a hypoplastic or absent odontoid process. Other notable medical problems include mild corneal clouding, joint contractures, and diffuse joint, muscle, and bone pain. Multiple surgeries have included bilateral hip replacement, gynecomastia reduction, gastric bypass, and cholecystectomy. There have been no reports of hearing problems, pulmonary or cardiac symptoms. At 37, he started enzyme replacement, which has resulted in improvement of restricted joint mobility.

#### Patient 4

Southern Chinese girl diagnosed at age 2 years (leukocyte GALNS 0.58 nmol/17 h/mg protein; reference range 42.3–441.96; *GALNS* variants c.106_111delCTGCTC / c.953 T > G). Study assessments were performed at 6.4 years of age. Physical examination at assessment demonstrated disproportionate short stature, with a height Z-score of − 6.0; mild coarse facial features, depressed nasal bridge, pectus carinatum, kyphosis, ulnar deviation of forearms, genu valgum, and severe joint hyperlaxity. Spinal magnetic resonance imaging (MRI) identified platyspondyly with cervicomedullary and T10 spinal canal stenosis, and absence of spinal instability despite incomplete ossification of the odontoid. Her echocardiogram identified normal left ventricular size and function with mild thickening of the mitral and aortic valve leaflets without regurgitation or stenosis. The aortic root absolute measurement was 18 mm (Z score + 1.20). She received rhGALNS from age 6.4 until age 8.2 with moderate reactions (hypotension, tachycardia, nausea, and emesis). ERT was discontinued at parental request due to arrest of linear growth and progression of genu valgum.

#### Patient 5

Hispanic male with *GALNS* pathogenic variants c.1156C > T and 32 kb deletion. At the time of study assessment, he was 18.3 years old and demonstrated disproportionate short stature, with a height Z-score of − 8.11; coarse facial features and depressed nasal bridge, and ulnar deviation of his forearms with wrist and ankle hyperlaxity. He required tracheostomy due to upper airway obstruction and tracheomalacia. He had been receiving rhGALNS ERT beginning at age 17.3 without any infusion-associated reactions.

#### Patient 6

Hispanic male diagnosed at 2.8 years of age (*GALNS* c.901G > T / c.1520G > T pathogenic variants) after presenting at 3 months of age with lumbar gibbus and subsequently developing disproportionate short stature. At study assessment, he was 3.87 years old and his height Z score was − 3.73. Physical examination identified frontal bossing, depressed nasal bridge, pectus carinatum, thoracolumbar kyphosis, global joint hyperlaxity, and genu valgum. Spinal MRI identified platyspondyly with moderate cervical canal stenosis, and hypoplastic dens without craniocervical instability. Echocardiogram identified thickening of mitral and aortic valve leaflets with trivial mitral valve regurgitation and normal aortic valve function. His aortic root was moderately dilated with a measurement of 21.6 mm (Z score + 3.28). He began receiving rhGALNS at age 3.2 ERT without reactions. Subsequently, he has required acetabuloplasty and femoral/tibial epiphyseodesis due to hip dysplasia and genu valgum.

#### Patient 7

Caucasian female who presented at 18 months of age with genu valgum and spinal kyphosis, and subsequently diagnosed with Morquio A at age 3 (deficient leukocyte GALNS enzyme; one pathogenic *GALNS* c.239C > T variant identified). Her echocardiogram, carotid ultrasound, and other study assessments were performed at age 8.5 years. Physical examination at assessment demonstrated a height Z score of − 6.77, disproportionate short stature, depressed nasal bridge, prominent pectus carinatum, severe wrist and ankle joint hyperlaxity, and genu valgum. Spinal MRI showed platyspondyly, craniocervical stenosis without instability, thoracic kyphosis, and lumbar hyperlordosis. Cardiac imaging identified trivial mitral and aortic valve regurgitation, and an aortic root measurement of 20.6 mm (Z score + 1.97). She has required bilateral hip acetabuloplasty and femoral/tibial epiphyseodesis. She had received investigational rhGALNS and declined to continue enzyme therapy.

#### Patient 8

She is the younger sister of patient 7 and was diagnosed at birth due to family history. She was 4.6 years old at time of study assessment, which identified a height Z score was − 2.17 with physical examination significant for disproportionate short stature, depressed nasal bridge, pectus carinatum, mild wrist / ankle joint hyperlaxity, thoracolumbar gibbus deformity, and genu valgum. Spinal MRI showed platyspondyly, and mild cervical spinal canal stenosis without instability. Echocardiogram identified thickening of the mitral and aortic valves without valve dysfunction. The aortic root measured 18 mm (Z score + 1.06). She has also undergone bilateral hip acetabuloplasty and femoral/tibial epiphyseodesis. She received investigational rhGALNS as an infant and declined to continue with enzyme therapy. At most recent clinical assessment, her short stature has worsened, and her current height Z score is − 6.34 at age 8.25 years, demonstrating a severe Morquio A phenotype like her older sister.

#### Patient 9

He is a Hispanic male initially misdiagnosed as spondyloepiphyseal dysplasia, subsequently identified to have Morquio A at 15 years of age after biochemical evaluation discovered elevated urinary KS and deficient fibroblast GALNS enzymatic activity. His study echocardiogram, carotid ultrasound, and other evaluations were performed at 25.9 years of age. Study evaluation was significant for disproportionate short stature (adult height 134.4 cm; approximate height Z score − 6.09), minimally coarse facial features, ulnar deviation of his forearms, mild joint hyperlaxity, and minimal genu valgum status post femoral/tibial epiphyseodesis. He had also undergone T10 – L3 spinal fusion due to kyphosis. Echocardiogram identified mild thickening of the mitral valve with neither mitral nor aortic valve dysfunction. His aortic arch could not be well-visualized due to habitus. He declined rhGALNS therapy.

#### Patient 10

He is a Hispanic male who was diagnosed at 3 years of age following identification of dysostosis multiplex and subsequent demonstration of urinary keratan sulfaturia with deficient fibroblast GALNS enzyme (7000 units; reference range > 40,000) and eventual demonstration of *GALNS* pathogenic variants (c.1156C > T / c.924 T > G + c.930G > C). Study assessments were performed at 22.26 years of age. Physical examination identified macrocephaly (56.5 cm), short stature (adult height 109.2 cm; Z score − 9.1), mild corneal clouding, coarse facial features, marked wrist and ankle joint laxity, genu valgum, brachydactyly, pectus carinatum, and thoracolumbar kyphosis. His echocardiogram was significant for normal mitral valve anatomy and function, but moderate aortic valve regurgitation, mild left ventricular dilatation (Left ventricular / LV mass index 170.1 g/m2), and aortic root dilatation (32.9 mm, Z-score + 4.95). He had undergone occipitocervical decompression and fusion, as well as bilateral hip osteotomies prior to study assessments. He has been treated with rhGALNS without reactions.

#### Patient 11

She is a Cuban/Caucasian female, first noted by parents at 2 years of age to have lumbar kyphosis. Subsequent spinal MRI suggested MPS, and biochemical evaluations identified elevated urinary KS, deficient fibroblast GALNS (0.05 units, reference range > 0.7), and eventual molecular confirmation of *GALNS* pathogenic mutations (c.776G > A / c.1520G > T). Study echocardiography, ultrasound, and examination occurred at 10.4 years of age. Study physical examination identified mild disproportionate short stature (height Z-score − 2.23), minimally coarse facial features, normal corneas, absence of pectus carinatum, and mild joint hyperlaxity. S-shaped curvature of the cervical-thoracic junction manifested with cord compression at age 11 and required decompression with spinal fusion. Her echocardiogram showed minimally redundant mitral and aortic valve leaflets without regurgitation or stenosis. Her aortic root measured 21 mm (Z-score + 0.43). She has been treated with intravenous rhGALNS without complications.

#### Patient 12

Hispanic male whose first notable symptom of Morquio A was pectus carinatum at age 2 years. After developing abnormal gait at 5 years of age, he was referred for clinical genetics evaluation and formally diagnosed with Morquio A at age 8. Urinary GAG excretion was elevated; his leukocyte GALNS activity was undetectable, and sequencing of *GALNS* identified c.281G > A / c.1156C > T pathogenic variants. Study assessments were performed at 16.1 years of age. Study physical exam demonstrated disproportionate short stature (height Z-score − 4.27), mild facial coarseness, pectus carinatum, thoracolumbar gibbus, forearm ulnar deviation, and hyperlaxity of joints. Mitral valve anatomy and function were normal, while he demonstrated trivial aortic valve regurgitation. Aortic root was mildly dilated, measuring 28.2 mm (Z-score + 2.23). He has been treated with intravenous rhGALNS without reactions.

### Correlation between genotype and phenotype

Patients 1 and 2 are homozygous for the mutation p.P77R (c.230C > G) in exon 2, which is associated with a severe phenotype due to a non-conservative change of proline to arginine in a conserved amino acid region of GALNS affecting its hydrophobic core [13, 14]. These patients show mildly thickened mitral valve and/or mildly thickened aortic valve with normal function. Patients 3 and 11 exhibit an attenuated phenotype based on their growth charts. Despite the difference in their age and gender, these patients have normal or minimally thickened mitral and aortic valves with normal function and normal aortic root Z score. Patient 3 is homozygous for the novel mutation p.R451S (c.1353 G > T) in exon 12 that is likely to be pathogenic with an *in-silico* score of 0.918 (sensitivity: 0.81; specificity: 0.94) [15]. Patient 11 has the allele c.776 G > A (p.R259Q, in exon 8), which is located on the surface of GALNS enzyme and gives an attenuated phenotype [13]. The other allele was c.1520 G > T (p.C507F) in exon 14 which has been classified as “likely disease-associated” mutation and has not been characterized in detail [16].

Patients 10 and 12 (22.3 and 16.1 years old, respectively) have severe phenotype of the disease with aortic valve pathology including cusp asymmetry or slightly thickened aortic valves with moderate regurgitation. In addition, both patients show a dilated aortic root. Their genotypes share the allele c.1156C > T (p.R386C) which is the most frequently reported *GALNS* mutation, located in exon 11 and associated with severe phenotype [14, 17, 18].

Patients 4 and 6, who were 6.4 and 3.9 years of age, have severe phenotype with severe heart disease evidenced by mitral and aortic valve pathology. Both have exon 9 mutations (p.M318R and p.G301C) known to impact protein folding and cause severe phenotype [13, 19–21].

Patients 7 and 8 (8.5 and 4.6 years old, respectively) are siblings with severe phenotype with mitral and valve pathology but with normal aortic root Z-score. They share the mutation p.S80L which affects the active site of GALNS enzyme [21–23]. The other allele has not been elucidated, but the Morquio A diagnosis is secure given patient 7’s low GALNS enzymatic activity.

## Discussion

This study of twelve patients with MPS IVA, or Morquio A disease, demonstrates a high prevalence of structural and functional abnormalities in their carotid arteries and left-sided cardiac valves. The small MPS IVA cohort and the cross-sectional nature of sonographic assessments represent limitations to this study; however, we have utilized the same approach for statistical analyses with other, similarly-sized MPS cohorts [11, 12].

### Carotid intima-media thickness

Mean cIMT of Morquio A patients was 0.56 mm, comparable to the mean cIMT of 0.56 mm found in 33 patients with other types of MPS (17 MPS I, 9 MPS II, 4 MPS IIIA, and 3 MPS VI), and 0.12 mm (26%) thicker than the mean cIMT of a large, healthy control cohort. ERT did not affect cIMT measurements in the Morquio A cohort. The mechanism of cIMT thickening in Morquio A is very similar to other MPS types: a combination of mechanical expansion caused by GAG storage, extracellular matrix proliferation, and recruitment of myofibroblasts and activated macrophages throughout arterial vasculature [12]. The in vivo cIMT findings corroborate the post-mortem examination of aortic intima and media from a 20-year old male with Morquio A disease, which identified infiltration of foamy GAG-laden macrophages and abundant accumulation of C6S within the intima-media matrix [8].

### Mitral and aortic valve disease

Hypertrophy of the decedent’s mitral and aortic valves, which contained large numbers of foam cells and inflammatory T-cell and macrophages has been reported [8]. Such findings correlate with the in vivo findings of our Morquio A cohort, which demonstrated a high proportion of patients with thickening of the mitral and aortic valves, and a smaller percentage who had valvular regurgitation. Age did not clearly correlate with severity of valve thickening or dysfunction. Valve thickening was observed in young and adult patients alike, and the three patients who did not have mitral valve thickening were 16.1, 22.3, and 38.4 years old. Young patients’ valve thickening may reflect greater Morquio A severity. Likewise, absence of valve thickening in the oldest patient reflects a more attenuated disorder. Despite this observation, we note that the 22.3-year-old who had a normal mitral valve also had the most dysplastic and regurgitant aortic valve. Thus, valve disease in Morquio A may also be simultaneously discordant and progressive (this patient’s aortic regurgitation worsened with age).

### Carotid hyper-elasticity

An unexpected finding of this study was markedly increased carotid elasticity in Morquio A patients. The Morquio A cohort had cCSC1 that was 43% and 64% greater than the control and non-Morquio A MPS cohorts, and a cIEM that was 33% and 54% reduced compared to the control and non-Morquio A MPS cohorts. This is counter to the significantly reduced carotid elasticity in patients with non-Morquio A MPS previously identified by our group [10–12]. Compliance or distensibility did not differ between Morquio A patients on or off ERT, but cIEM was higher in the ERT-treated group after adjustment for sex, age, and height. Whether this difference is an effect of treatment will require additional, longitudinal assessments. Elastin, a highly abundant protein within the arterial media, is arranged in tightly coiled laminae and allows arteries to withstand blood pressure oscillations throughout the cardiac cycle. Attenuated, fragmented arterial elastin laminae have been observed in MPS types I, VI, and VII patients and animal models [12, 24], a result of proteolysis by cathepsins and matrix metalloproteinases secreted by GAG-activated macrophages [25]. We hypothesized that reduced carotid elasticity in MPS I, II, IIIA, and VI patients was a consequence of the destruction in arterial elastin. Precedence for this hypothesis comes from Marfan syndrome patients, where disarrayed and fragmented arterial elastin fibrils are a cardinal feature, giving rise to reduced carotid compliance and distensibility and ultimately life-threatening aortic root dissection [26]. Since elastin attenuation and fragmentation was observed in Yasuda’s Morquio A patient [8], we expected the Morquio A cohort to demonstrate abnormally low carotid elasticity compared to our other MPS cohorts.

Despite the unexpectedly increased carotid elasticity in the Morquio A cohort, the cohort’s high prevalence of aortic root dilatation (56%), comparable to the 69% (11 of 16) prevalence in an independently ascertained Morquio A cohort [27] and a high prevalence in older Morquio A patients [7] comprise evidence that pathology of arterial elastin in Morquio A has clinical significance. While no Morquio A patient has yet been reported to experience aortic dissection, improved life spans of MPS IVA patients necessitate education for families and physician providers to recognize and promptly seek medical intervention if dissection symptoms develop. An additional potential step, mirroring aortic root management in Marfan syndrome, would be to determine what magnitude of aortic root dilatation, if any, would warrant elective surgical correction. The existence of multiple co-morbidities in Morquio A, notably airway access challenges [28], redundancy and malacia of the tracheobronchial tree [29], and risk of watershed spinal ischemia [30], result in an extremely high-risk surgery. Thus, the authors are reluctant to recommend any aortic root surgery until further natural history studies document the occurrence of aortic root dissection in Morquio A.

Carotid hyper-elasticity in the context of arterial elastin fragmentation in Morquio A seems incongruous, but may uniquely illuminate the function GAG-bearing proteoglycans (PGs) in arterial health. Differences in GAG species accumulated in Morquio A (KS and C6S), compared to MPS types I / II / VI (heparan and dermatan sulfates; HS and DS, respectively), may be the key to understanding the stark differences in carotid function between Morquio A and other types of MPS. GAGs represent 1–2% of arterial dry weight in healthy individuals [31, 32]. Attached to PGs and secreted into the extracellular matrix, they form complexes with elastin, collagen, and other fibrillar proteins that generate viscoelastic properties required to maintain arterial integrity through a lifetime of cardiac cycles. Alterations in balance between KS, C6S, HS, and DS-bearing PGs may alter arterial elastic properties independent of elastin content. Indeed, versican, a C6S-PG, is known to accumulate in early intimal thickening, large atherosclerotic lesions, and areas of vascular injury and serves as a ligand for inflammatory macrophage adhesion and chemokines [33]. Short of atherosclerosis, many of these findings are observed in Morquio A patients and model systems, making such versican and other KS/C6S-bearing PGs the focal point of MPS vascular pathology investigation. Perhaps these investigations will also explain joint hyperlaxity only observed in Morquio A patients, a contrast to the joint stiffness and contractures in all other MPS types. Additional studies are in progress to better document the cardiovascular natural history in Morquio A patients, and to compare with great detail the alterations and downstream effects of arterial KS/C6S storage in the Morquio A model system, versus arterial HS/DS storage in MPS I model system.

## Conclusions

Carotid hyper elasticity, increased thickening of left-sided heart valves, and increased cIMT constitute most of the structural and functional abnormalities observed in this cohort of 12 Morquio A patients. Since cardiac lesions worsen with time, patients should be evaluated periodically by their cardiologist. Adding carotid ultrasonography with evaluation of cIMT and vessel elasticity to the diagnostic toolbox will aid in understanding Morquio A progression and evaluating current and novel treatment strategies.

## Methods

### Study aim, design, and settings

The study aimed to compare echocardiographic findings and carotid intima-media thickness and stiffness in individuals with Morquio A disease, other MPS types, and unaffected controls utilizing a prospective, case-control design. The study took place at Children’s Hospital of Orange County, Saint Louis University, and the University of Minnesota.

### Human subjects

Studies were conducted following informed consent / assent (CHOC IRB#131107, SLU IRB#24454). Morquio A was diagnosed based on reduced GALNS enzyme activity of ≤5% the normal level in plasma, fibroblasts or leukocytes, and/or via *GALNS* molecular testing demonstrating two or more pathogenic mutations. All Morquio A patients followed clinically at CHOC and Saint Louis University were eligible for the study, and all enrolled; control patients were ascertained at the University of Minnesota. We classified disease severity by patient height, with attenuated disease classified as ≥90th centile height isopleth, and severe disease as <90th centile height isopleth for Morquio A patients [34]. Patient urine was collected after obtaining informed patient consent or guardian assent. Enzyme activity in patient samples was compared to activity in urine samples from five healthy controls to confirm Morquio A diagnosis. Chart review was conducted for the 12 Morquio A patients, and relevant summaries of clinical, biochemical, radiographic, and molecular diagnostics evaluations are summarized in Table 1.

### Carotid ultrasound

Ultrasonographers with at least 7 years of experience examined patients, assisted by a senior technologist. All exams were performed with a General Electric Logiq E9 machine (GE Healthcare, Waukesha, WI) and linear 9 mHz transducer. A standard automated blood pressure monitor was used with pediatric cuff. Subjects were placed supine without a pillow and with legs uncrossed. Subjects were then asked to extend their neck and tilt their head back and to the right to expose the left common carotid artery. Subjects were asked to close their mouths and to not speak or swallow during the exam. An automatic blood pressure cuff was applied to the right upper arm. The ultrasound transducer was placed approximately 10 mm proximal to the bifurcation of the internal and external carotid arteries in the longitudinal plane. The transducer was manipulated to optimize visualization of the far wall intima-media thickness. Presets were adjusted to carotid mode; parameters such as focal zone placement, gain, and time gain compensation were optimized by the examiner on a case by case basis. The automatic blood pressure machine was engaged. The technologist then took a 10-s cine series while blood pressure was obtained. Exams were saved in the Picture Archiving and Communication System (PACS) and analyzed at a central facility (University of Minnesota). Of note, Patient 2 underwent two carotid ultrasounds at 7.0 and 8.4 years of age. Data from both time points are reported and incorporated in statistical analyses.

### Carotid structure and function definitions

Carotid intima-media thickness (cIMT), cross-sectional distensibility (cCSD), cross-sectional compliance (cCSC1), and incremental elastic modulus (cIEM) [35] were calculated utilizing Vascular Research Tools 5, Medical Imaging Application, LLC, Coralville, IA, USA. Although multiple technicians were involved in collection of carotid ultrasounds, this software suite automates the quantification of the parameters and is operator-independent, minimizing any variability between technicians [35].

cCSD is defined by the percentage change in area of the carotid lumen from diastole to systole and is defined by the equation [35]:

$$ cCSD=\frac{sD^2-{dD}^2}{dD^2}\bullet 100\% $$

where sD indicates the maximum carotid lumen diameter during systole, and dD is the minimum carotid lumen diameter during diastole. A higher vessel cCSD indicates increased elasticity / reduced stiffness; a lower vessel cCSD corresponds to reduced elasticity / increased stiffness.

cCSC1, which has a unit of mm^2^ / mm Hg, is defined by the relative change in carotid lumen area from diastole to systole per unit change in blood pressure and is defined by the equation:

$$ cCSC1=\frac{\pi\ \left({sD}^2-{dD}^2\right)}{4\bullet \left( SBP- DBP\right)} $$

where SBP and DBP refer to systolic and diastolic blood pressure, respectively. Another way to conceptualize cCSC1 is the increase in carotid lumen area for each increase in blood pressure of 1 mmHg. Similar to cCSD, increasing cCSC1 represents increased elasticity / reduced stiffness (more change in carotid area for a given change in blood pressure), and vice versa.

Finally, cIEM represents the carotid elasticity constant. Increased cIEM indicates more difficulty to distend the vessel and therefore reduced elasticity / increased stiffness, and vice versa. The equation for cIEM, which has the unit of mm Hg, is:

$$ cIEM=\frac{3\bullet \left(1+\frac{sD^2}{dD^2}\right)}{cCSC1} $$

### Apparatus and sample preparation

The chromatographic system consists of an HP1100 system (Agilent Technologies, USA) and a Hypercarb column (2.0 mm i.d. 150 mm, 5 μm, Thermo Electron, USA). An API-4000 mass spectrometer (Applied Biosystems) equipped with a turbo ionspray ion source was used. Stock solution of Galß1,4GlcNAc(6S) (500 μg/ml), ΔDiHS-0S (100 μg/ml), ΔDiHS-NS (10 μg/ml), ΔDi-4S (100 μg/ml), ΔDi-6S (100 μg/ml) (Seikagaku, Tokyo, Japan), and internal standard (IS-chondrosine) (50 μg/ml) (Glycosyn, New Zealand) were prepared in water. Urine was centrifuged and supernatants digested overnight with 1 mU of chondroitinase b, 1 mU heparitinase, and 1 mU keratanase II (Seikagaku, Tokyo, Japan) in buffer solution (1% BSA, 50 mM Tris-HCl, 5 mg/ml Chondrosine). Recovered samples were analyzed by the liquid chromatography-tandem mass spectrometry (LC-MS/MS) system and normalized by creatinine concentration in urine. Disaccharide GAG concentrations were calculated by Analyst 1.5.1 software (AB SCIEX, Foster City, CA).

### Statistical analysis

Descriptive summaries were tabulated separately by disease group. These included the mean and standard deviation for continuous variables and frequency for categorical variables. One MPS IVA patient was measured at two time points, 1.35 years apart: both measurements were included in analysis. Confidence intervals for proportions were computed by inverting the score test. Linear regression was used to evaluate differences in mean values between groups adjusting for age, sex, and height with robust variance estimation used for confidence intervals and *P*-values. Regression to evaluate ratios of means were evaluated similarly, but with a log-link. Statistical analyses were performed using R v3.5.1 [36].

## Data Availability

The datasets used and analyzed during the current study are available from the corresponding author on reasonable request.

## Abbreviations

C6S: Chondroitin-6-sulfate
cCSC: Carotid Cross-Sectional Compliance
cCSD: Carotid Cross-Sectional Distensibility
cIEM: Carotid Incremental Elastic Modulus
cIMT: Carotid Intima-Media Thickness
DBP: Diastolic blood pressure
DS: Dermatan sulfate
ERT: Enzyme Replacement Therapy
GAGs: Glycosaminoglycans
GALNS: N-acetylgalactosamine-6-sulfate sulfatase
HS: Heparan sulfate
KS: Keratan sulfate
LC-MS/MS: Liquid chromatography – tandem mass spectrometry
LV: Left ventricular
mm Hg: Millimeters of mercury
MPS: Mucopolysaccharidoses
MRI: Magnetic resonance imaging
rhGALNS: Recombinant Human N-acetylgalactosamine-6-sulfate sulfatase
SBP: Systolic blood pressure
